# Bidirectional association between aortic dissection and atrial fibrillation: Findings from a huge national database

**DOI:** 10.1002/clc.23223

**Published:** 2019-07-10

**Authors:** Wei‐Syun Hu, Cheng‐Li Lin

**Affiliations:** ^1^ School of Medicine, College of Medicine China Medical University Taichung Taiwan; ^2^ Division of Cardiovascular Medicine, Department of Medicine China Medical University Hospital Taichung Taiwan; ^3^ Management Office for Health Data China Medical University Hospital Taichung Taiwan

**Keywords:** aortic dissection, atrial fibrillation, cohort

## Abstract

**Objective:**

To explore the link between aortic dissection (AD) and atrial fibrillation (AF).

**Methods:**

Using the National Health Insurance Research Database (NHIRD), cohorts were constructed for evaluating the incidence of AF in patients with AD (study 1) and the incident AD among AF patients (study 2) based on propensity matching analysis. Cox proportion hazard regression models were used to examine the effect of AD on the risk of AF, shown as hazard ratios (HRs) with 95% confidence intervals (CIs). Similar statistical procedures were used for study 2.

**Results:**

The study 1 consisted of 11 813 patients in the AD cohort and 11 813 controls in the non‐AD cohort and the study 2 consisted of 190 494 patients in the AF cohort and 190 494 controls in the non‐AF cohort. The overall incidence density of AF was 1.32‐fold higher in the AD cohort than in the non‐AD cohort (11.1 and 8.3 per 1000 person‐years), with an adjusted HR (aHR) of 1.74 (95% CI = 1.53‐1.98). The AF cohort had 1.18‐fold higher incidence of AD than the non‐AF cohort (0.55 vs 0.47 per 1000 person‐years), with an aHR of 1.24 (95% CI = 1.07‐1.44).

**Conclusions:**

Bidirectional association between AD and AF was shown for the first time in this study.

## 1 INTRODUCTION

Aortic dissection (AD) is a life threating disease once left undiagnosed or untreated.^1^, ^2^ The phenomenon of AD presenting with atrial fibrillation (AF) has indeed been discussed previously^3^, ^4^, ^5^, ^6^, ^7^, ^8^, ^9^; in case of subclinical AD, AF may occur and be a sign of alert. For clinicians who care for patients with AF, it is well known that stroke, heart failure, and death are common AF complications.^10^, ^11^, ^12^ To date, whether there is an increased risk of AD in patients with AF remained unknown.

To provide additional evidence linking AD and AF from the view point of clinical aspect, investigation on the relationship between AF and AD might be thoughtful. Hence, we sought to utilize the Taiwanese national dataset to describe the incidence of AF in patients with AD and the incidence of AD in patients with AF, using propensity score methods, multivariate controlling and combining a large number of comorbidities in our analysis to explore the link between AD and AF.

## 2 METHODS

### 2.1 Data source

We used the National Health Insurance Research Database (NHIRD) of the National Health Insurance (NHI) program in Taiwan to conduct this retrospective nationwide cohort study. The NHI program was established by the Taiwanese government on March 1, 1995, and it covered more than 99% of the 23.74 million residents in Taiwan. In this retrospective cohort study, the history of disease diagnosis was obtained from inpatient files, with data available from 1996 to 2011. The diagnoses in Taiwan NHI were coded according to the International Classification of Disease, Ninth Revision, Clinical Modification (ICD‐9‐CM). The Research Ethics Committee of China Medical University and Hospital in Taiwan approved the study (CMUH‐104‐REC2‐115‐R3).

### 2.2 Sampled participants

For study 1, we identified patients aged 18 or older years with AD diagnosed between 2000 and 2010 (ICD‐9‐CM codes 441.0) and control individuals without AD. The index date for control patients was randomly appointed a month and day with the same index year of the matched AD cases. We defined the diagnosed date of AD as the index date for each patient. We excluded patients with a diagnosis of AF (ICD‐9‐CM codes 427.31) at baseline and those with incomplete medical records information. Patients in the AD and non‐AD cohorts were selected by 1:1 matching based on a propensity score.^13^ The propensity score was calculated using a logistic regression model to estimate the probability of the AD status assignment, based on the baseline variables including year of AD diagnosis, sex, age, and comorbidities of hypertension, diabetes mellitus (DM), hyperlipidemia, coronary heart disease (CHD), heart failure (HF), chronic obstructive pulmonary disease (COPD), peripheral artery disease (PAD), chronic kidney disease (CKD), hyperthyroidism, sleep disorders, gout, cerebrovascular disease, chronic liver disease, cancer, asthma, peptic ulcer disease, and vavular heart disease (VHD). For study 2, patients aged 18 or older years with AF diagnosed between 2000 and 2010 and control individuals without AF were identified. Patients suffering from AD at the baseline and those with missing medical records information were excluded. For each AF identified, controls were selected and matched by propensity score under the same exclusion criteria. The propensity score was calculated using a logistic regression model to estimate the probability of the AF status assignment, based on the baseline variables including year of AF diagnosis, sex, age, and comorbidities of hypertension, DM, hyperlipidemia, CHD, HF, COPD, PAD, CKD, hyperthyroidism, sleep disorders, gout, cerebrovascular disease, chronic liver disease, cancer, asthma, peptic ulcer disease, and VHD.

### 2.3 Outcome

Subjects in the study 1 were followed until the diagnosis of AF or until withdrawal from the NHI program or death, or December 31, 2011. Subjects in the study 2 were followed until the diagnosis of AD or until withdrawal from the NHI program or death, or December 31, 2011.

### 2.4 Statistical analysis

For study 1, the distributions of the sex, age, and comorbidities were compared between the AD cohort and the non‐AD cohort, and the differences were examined using the standardized mean difference (SMD). A SMD of ≤0.10 indicates a negligible difference between the two cohorts. The overall, sex‐, age‐, comorbidity‐specific, and follow‐up period incidence densities rate of AF (per 1000 person‐years, PY) were measured for each cohort. Univariable and multivariable Cox proportion hazard regression models were used to examine the effect of AD on the risk of AF, shown as hazard ratios (HRs) with 95% confidence intervals (CIs). The multivariable‐adjusted models included covariates that were not adequately balanced in Tables 1 and 3 (standardized difference > 0.1). The cumulative incidence curve of AF was computed using the Kaplan‐Meier method and the differences between both cohorts were examined using the log‐rank test. Similar data analysis procedures were performed to calculate the incidence density rates of AD (per 1000 person‐years, PY) and HRs (95% CIs) for the AF and non‐AF cohorts in the study 2. Data analyses were conducted using statistical package SAS (Version 9.4, SAS Institute Inc., Carey, North Carolina). A two‐tailed *P* value < .05 was considered statistically significant.

**Table 1:** Demographic characteristics and comorbidities in patients with and without aortic dissection

	Aortic dissection				
	No (N=11813)		Yes (N=11813)		
Variables	n	%	n	%	Standardized mean differences§
Sex					
Female	3797	32.1	3375	28.6	0.08
Male	8016	67.9	8438	71.4	0.08
Age, years					
20–49	1488	12.6	2201	18.6	0.17
50–64	3015	25.5	3683	31.2	0.13
≥ 65	7310	61.9	5929	50.2	0.24
Mean (SD) ^†^	67.5	14.5	63.9	14.6	0.25
Comorbidity					
Hypertension	8598	72.8	8357	70.7	0.05
Diabetes mellitus	1964	16.6	1505	12.7	0.05
Hyperlipidemia	1330	11.3	1149	9.73	0.05
CHD	2994	25.3	3078	26.1	0.02
Heart failure	1048	8.87	1141	9.66	0.03
COPD	1205	10.2	1138	9.63	0.02
PAD	332	2.81	426	3.61	0.05
CKD	457	3.87	416	3.52	0.02
Hyperthyroidism	90	0.76	50	0.42	0.04
Sleep disorders	337	2.85	279	2.36	0.03
Gout	1013	8.58	979	8.29	0.01
Cerebrovascular disease	2328	19.7	2290	19.4	0.01
Chronic liver disease	987	8.36	716	6.06	0.09
Cancer	764	6.47	509	4.31	0.10
Asthma	735	6.22	607	5.14	0.05
Peptic ulcer disease	2345	19.9	1907	16.1	0.10
VHD	1207	10.2	1581	13.4	0.10

§ A standardized mean difference of ≤0.10 indicates a negligible difference between the two cohorts.

CHD, coronary heart disease; CKD, chronic kidney disease; COPD, chronic obstructive pulmonary disease; DM, diabetes mellitus; PAD, peripheral artery disease; VHD, valvular heart disease.

## 3 RESULTS

### 3.1 Study 1

**Figure 1:**
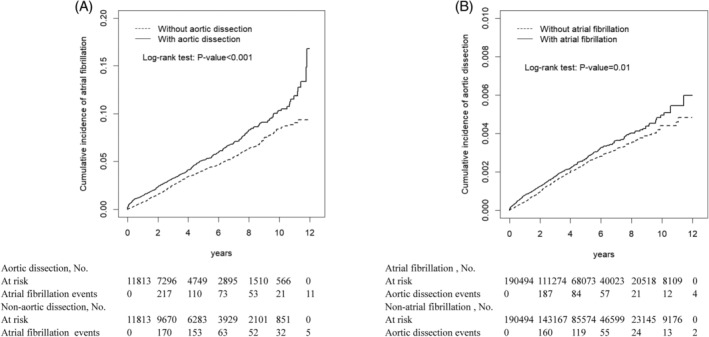
A, Cumulative incidence of atrial fibrillation (AF) for patients with (solid line) and without (dashed line) aortic dissection. B, Cumulative incidence of aortic dissection for patients with (solid line) and without (dashed line) AF

The study 1 consisted of 11 813 patients in the AD cohort and 11 813 controls in the non‐AD cohort (Table 1). Men represented the majority of the study cohorts (71.4% vs 67.9%) and over a half of study population were more than 65 years old. The AD cohort were slightly younger than the non‐AD cohort. The average follow‐up duration was 3.71 ± 3.19 years for the AD cohort and 4.85 ± 2.99 years for the non‐AD cohort. Figure 1A shows that the cumulative incidence of AF was higher in the AD cohort than in the non‐AD cohort (the log‐rank test *P* < .001) after 12 years of follow‐up.

The overall incidence density of AF was 1.32‐fold higher in the AD cohort than in the non‐AD cohort (11.1 and 8.3 per 1000 person‐years), with an adjusted HR (aHR) of 1.74 (95% CI = 1.53‐1.98) after controlling for age (Table 2).

**Table 2:** Incidence and hazard ratios of atrial fibrillation for aortic dissection cohort compared to non‐aortic dissection cohort by demographic characteristics, comorbidity and follow‐up year

	Aortic dissection							
	No (N = 11 813)			Yes (N = 11 813)				
Variables	Event	person‐years	Rate^#^	Event	Person‐years	Rate^#^	Crude HR (95% CI)	Age‐adjusted HR (95% CI)
Total	475	57 237	8.3	485	43 870	11.1	1.32 (1.17, 1.50)***	1.74 (1.53, 1.98)***
Sex								
Female	165	18 081	9.13	161	12 147	13.3	1.44 (1.16, 1.79)**	1.76 (1.42, 2.20)***
Male	310	39 157	7.92	324	31 723	10.2	1.29 (1.10, 1.50)**	1.73 (1.48, 2.03)***
Age, years								
20‐49	9	8347	1.08	34	10 282	3.31	3.06 (1.47, 6.39)**	
50‐64	55	16 269	3.38	90	15 555	5.79	1.71 (1.22, 2.39)**	
≥65	411	32 620	12.6	361	18 033	20.0	1.59 (1.38, 1.83)***	
Comorbidity^a^								
No	34	6528	5.21	40	4820	8.30	1.59 (1.01, 2.52)*	3.44 (2.11, 5.59)***
Yes	441	50 709	8.70	445	39 050	11.4	1.30 (1.14, 1.49)***	1.64 (1.43, 1.87)***
Hypertension	383	39 955	9.59	349	31 726	11.0	1.14 (0.99, 1.32)	1.51 (1.30, 1.74)***
DM	96	8260	11.6	75	4733	15.9	1.35 (1.00, 1.83)	1.51 (1.11, 2.04)**
Hyperlipidemia	56	6134	9.13	54	4182	12.9	1.39 (0.96, 2.02)	1.51 (1.04, 2.20)*
CHD	180	13 295	13.5	184	10 628	17.3	1.27 (1.03, 1.56)*	1.51 (1.23, 1.85)***
Heart failure	86	3622	23.7	96	2861	33.6	1.41 (1.05, 1.88)*	1.74 (1.29, 2.33)***
COPD	95	4700	20.2	80	3056	26.2	1.28 (0.95, 1.73)	1.32 (0.98, 1.78)
PAD	19	1257	15.1	23	1203	19.1	1.23 (0.67, 2.26)	1.49 (0.80, 2.76)
CKD	22	1632	13.5	23	1171	19.7	1.45(0.81, 2.61)	1.61(0.90, 2.90)
Hyperthyroidism	5	480	10.4	4	181	22.1	1.96 (0.52, 7.36)	1.58 (0.40, 6.22)
Sleep disorders	11	1345	8.18	14	994	14.1	1.73 (0.78, 3.81)	1.99 (0.90, 4.40)
Gout	53	4344	12.2	52	3369	15.4	1.23 (0.84, 1.81)	1.45 (0.98, 2.13)
Cerebrovascular disease	101	9646	10.5	116	6449	18.0	1.70 (1.30, 2.22)***	2.00 (1.53, 2.61)***
Chronic liver disease	38	4336	8.76	32	2236	14.3	1.64 (1.02, 2.63)*	1.63 (1.02, 2.61)*
Cancer	26	2771	9.38	23	1315	17.5	1.87 (1.07, 3.28)*	1.80 (1.02, 3.16)*
Asthma	54	3129	17.3	49	1870	26.2	1.48 (1.00, 2.18)*	1.41 (0.95, 2.07)
Peptic ulcer disease	99	10 366	9.55	98	6068	16.2	1.67 (1.26, 2.21)***	1.78 (1.34, 2.35)***
VHD	85	5139	16.5	96	5856	16.4	1.00 (0.75, 1.34)	1.52 (1.13, 2.06)**
Follow‐up year								
≦1	86	11 454	7.51	144	9468	15.2	1.98 (1.52, 2.59)***	2.44 (1.86, 3.20)***
2‐3	168	19 105	8.79	134	14 666	9.14	1.04 (0.83, 1.31)	1.41 (1.12, 1.78)**
4‐5	106	12 684	8.36	94	9584	9.81	1.17 (0.89, 1.55)	1.58 (1.19, 2.09)**
>5	115	13 994	8.22	113	10 152	11.1	1.36 (1.05, 1.76)*	1.82 (1.40, 2.37)***

Rate^#^, incidence rate per 1000 person‐years;

Abbreviations: CHD, coronary heart disease; CI; confidence interval; CKD, chronic kidney disease; COPD, chronic obstructive pulmonary disease; DM, diabetes mellitus; HR, hazard ratio; PAD, peripheral artery disease; VHD, valvular heart disease.

a Patients with any comorbidity of hypertension, diabetes mellitus, hyperlipidemia, CHD, heart failure, COPD, PAD, CKD, hyperthyroidism, sleep disorders, gout, cerebrovascular disease, chronic liver disease, cancer, asthma, peptic ulcer disease, and VHD were defined as the comorbidity group.

* *P* < .05

** *P* < .01

*** *P* < .001.

The incidence density and risk of AF were compared in the AD cohort and the non‐AD cohort regarding several variables including sex, age, with or without comorbidity, individual comorbidity and follow‐up period. The risk of AF in AD patients was also significantly higher than that of the non‐AD cohort in most stratified analysis (except for with comorbidity of COPD, PAD, CKD, hyperthyroidism, sleep disorders, gout, and asthma).

### 3.2 Study 2

The study 2 consisted of 190 494 patients in the AF cohort and 190 494 controls in the non‐AF cohort (Table 3). Both cohorts had more men (54.9% vs 55.3%) and more than 75% of the study population were aged ≥ 65 years. The average follow‐up duration was 3.47 years for the AF cohort and 4.19 years for the non‐AF cohort.

**Table 3:** Demographic characteristics and comorbidities in patients with and without atrial fibrillation

	Atrial fibrillation				
	No (N = 190 494)		Yes (N = 190 494)		
Variables	n	%	n	%	Standardized mean difference
Sex					
Female	85 117	44.7	85 883	45.1	0.01
Male	105 377	55.3	104 611	54.9	0.01
Age, years					
20‐49	6981	3.66	10 398	5.46	0.09
50‐64	25 770	13.5	29 875	15.7	0.06
≥65	157 743	82.8	150 221	78.9	0.10
Mean (SD)^a^	74.7	11.6	73.5	12.6	0.001
Comorbidity					
Hypertension	126 175	66.2	109 709	57.6	0.18
DM	59 551	31.3	50 827	26.7	0.18
Hyperlipidemia	27 603	14.5	22 241	11.7	0.08
CHD	77 171	40.5	74 482	39.1	0.03
Heart failure	47 807	25.1	63 957	33.6	0.19
COPD	38 867	20.4	39 788	20.9	0.01
PAD	5222	2.74	5536	2.91	0.01
CKD	10 819	5.68	10 466	5.49	0.01
Hyperthyroidism	4267	2.24	4222	2.22	0.002
Sleep disorders	6835	3.59	5904	3.10	0.03
Gout	16 735	8.79	15 320	8.04	0.03
Cerebrovascular disease	69 200	36.3	62 115	32.6	0.08
Chronic liver disease	19 120	10.0	15 346	8.06	0.07
Cancer	16 485	8.65	12 524	6.57	0.08
Asthma	21 261	11.2	20 910	11.0	0.01
Peptic ulcer disease	44 930	23.6	38 175	20.0	0.09
VHD	24 981	13.1	30 395	16.0	0.08

Abbreviations: CHD, coronary heart disease; CKD, chronic kidney disease; COPD, chronic obstructive pulmonary disease; DM, diabetes mellitus; PAD, peripheral artery disease; VHD, valvular heart disease.

a A standardized mean difference of ≤0.10 indicates a negligible difference between the two cohorts.

Figure 1B shows that the cumulative incidence of AD was higher in the AF cohort than in the non‐AF cohort (the log‐rank test *P* = .01) after 12 years of follow‐up. The AF cohort had 1.18‐fold higher incidence of AD than the non‐AF cohort (0.55 vs 0.47 per 1000 person‐years), with an aHR of 1.24 (95% CI = 1.07‐1.44) (Table 4). The sex‐specific AD risk for the AF cohort relative to the non‐AF cohort was significantly higher for women (aHR = 1.37; 95% CI = 1.08‐1.75). The age‐specific AD risk for the AF cohort relative to the non‐AF cohort was higher for the aged 50 to 64 group (aHR = 1.56; 95% CI = 1.06‐2.28) and for the aged ≥65 group (aHR = 1.19; 95% CI = 1.01‐1.40). Among the comorbid subjects, patients with AF had a higher risk of AD compared to the non‐AF cohort (aHR = 1.20 for hypertension; aHR = 1.47 for diabetes mellitus; aHR = 1.67 for chronic liver disease; aHR = 1.37 for peptic ulcer disease). In the first year of follow‐up, the AF cohort had a higher risk of AD compared with the non‐AF cohort (aHR = 1.78, 95% CI = 1.34‐2.36).

**Table 4:** Incidence and hazard ratios of aortic dissection for atrial fibrillation (AF) cohort compared to non‐AF cohort by demographic characteristics, comorbidity and follow‐up year

	Atrial fibrillation							
	No (N = 190 494)			Yes (N = 190 494)				
Variables	Event	person‐years	Rate^#^	Event	person‐years	Rate^#^	Crude HR (95% CI)	Adjusted HR^b^ (95% CI)
Total	373	799 500	0.47	365	662 905	0.55	1.18 (1.02, 1.36)**	1.24 (1.07, 1.44)**
Sex								
Female	130	363 359	0.36	140	296 615	0.47	1.30 (1.03, 1.66)*	1.37 (1.08, 1.75)*
Male	243	436 141	0.56	225	366 290	0.61	1.11 (0.92, 1.33)	1.19 (0.99, 1.44)
Age, years								
20‐49	8	36 393	0.22	11	56 431	0.19	0.91 (0.37, 2.27)	0.91 (0.36, 2.29)
50‐64	43	129 812	0.33	70	141 811	0.49	1.49 (1.02, 2.18)*	1.56 (1.06, 2.28)*
≥65	322	633 295	0.51	284	464 663	0.61	1.19 (1.02, 1.40)*	1.19 (1.01, 1.40)*
Comorbidity^a^								
No	22	827 950	0.27	11	56 404	0.20	0.76 (0.37, 1.57)	1.02 (0.48, 2.19)
Yes	351	716 751	0.49	354	606 501	0.58	1.19 (1.02, 1.38)*	1.23 (1.06, 1.43)**
Hypertension	291	502 799	0.58	254	356 329	0.71	1.23 (1.04, 1.45)*	1.20 (1.01, 1.42)*
DM	74	222 088	0.33	70	152 216	0.46	1.38 (1.00, 1.92)	1.47 (1.06, 2.05)*
Hyperlipidemia	64	110 929	0.58	52	78 388	0.66	1.15 (0.79, 1.65)	1.26 (0.87, 1.82)
CHD	187	309 199	0.61	176	254 405	0.69	1.14 (0.93, 1.40)	1.19 (0.97, 1.47)
Heart failure	102	166 921	0.61	125	192 255	0.65	1.06 (0.82, 1.38)	1.15 (0.88, 1.50)
COPD	92	136 483	0.67	79	101 140	0.78	1.15 (0.85, 1.56)	1.16 (0.86, 1.58)
PAD	14	17 129	0.82	13	13 384	0.97	1.17 (0.55, 2.49)	1.22 (0.56, 2.62)
CKD	17	30 967	0.55	14	22 952	0.61	1.13 (0.56, 2.30)	1.22 (0.60, 2.49)
Hyperthyroidism	8	19 883	0.40	2	18 760	0.11	0.28 (0.06, 1.29)	0.34(0.07, 1.64)
Sleep disorders	14	25 498	0.55	13	17 828	0.73	1.33 (0.62, 2.83)	1.40(0.65, 2.99)
Gout	56	62 697	0.89	34	47 656	0.71	0.80 (0.52, 1.22)	0.82(0.54, 1.27)
Cerebrovascular disease	145	264 166	0.55	119	183 467	0.65	1.18 (0.93, 1.51)	1.23 (0.97, 1.58)
Chronic liver disease	34	75 110	0.45	32	44 747	0.72	1.58 (0.98, 2.57)	1.67 (1.03, 2.71)*
Cancer	22	55 652	0.40	15	26 454	0.57	1.40 (0.73, 2.71)	1.40 (0.73, 2.71)
Asthma	43	79 031	0.54	30	57 735	0.52	0.94 (0.59, 1.51)	0.98 (0.61, 1.57)
Peptic ulcer disease	104	166 949	0.62	93	107 042	0.87	1.37 (1.04, 1.82)	1.37 (1.04, 1.82)*
VHD	56	98 491	0.57	78	106 329	0.73	1.29 (0.91, 1.81)	1.34 (0.95, 1.90)
Follow‐up year								
≤1	81	180 323	0.45	128	159 000	0.81	1.77 (1.34, 2.34)***	1.78 (1.34, 2.36)***
2‐3	153	283 691	0.54	108	225 350	0.48	0.89 (0.70, 1.14)	0.96 (0.75, 1.23)
4‐5	79	172 659	0.46	65	137 604	0.47	1.03 (0.74, 1.43)	1.11 (0.79, 1.55)
>5	60	162 827	0.37	64	140 951	0.45	1.24 (0.87, 1.76)	1.36 (0.95, 1.95)

*Note*: Rate^#^, incidence rate per 1000 person‐years.

Abbreviations: CHD, coronary heart disease; CI, confidence interval; CKD, chronic kidney disease; COPD, chronic obstructive pulmonary disease; DM, diabetes mellitus; HR, hazard ratio; PAD, peripheral artery disease; VHD, valvular heart disease.

a Patients with any comorbidity of hypertension, diabetes mellitus, hyperlipidemia, CHD, heart failure, COPD, PAD, CKD, hyperthyroidism, sleep disorders, gout, cerebrovascular disease, chronic liver disease, cancer, asthma, peptic ulcer disease, and VHD were defined as the comorbidity group.

b Model was adjusted for age, and comorbidities of hypertension, diabetes mellitus, and heart failure.

* *P* < .05

** *P* < .01

*** *P* < .001.

## 4 DISCUSSION

Using Taiwan national cohort claims data, the authors addressed for the first time the bidirectional association between AD and AF. Considering that there is no previous large scale report on the association between AD and AF to date, this is indeed data that could be helpful in the understanding and management of the growing population of adults with AD and AF.

The topic of secondary AF has received increasing attention as prior anecdotal beliefs that AF resolved after resolution of acute illness triggers have yielded to evidence suggesting high AF recurrence, morbidity, and mortality after secondary AF.^14^ Patients with AD complicated AF might have an increased risk of AF‐associated adverse events resulting in premature mortality.^10^, ^11^, ^12^ The two pathologies, AD and AF, are essentially different diseases. It is possible that before the development of AF in AD, there may be several factors that are also considered to be a cause of AF incidence. However, we adopted a propensity score‐matching analysis and multivariate adjustment to minimize these biases and the results were statistically true,^13^ and we observed that such association was stronger in those without comorbidity, implying that the development of AF in patients with AD might be independent of comorbidities.

Identification of AD is of importance in patients with AF because of high risk of death from AD.^1^, ^2^ The current study showed that the risk ratio was highest among women, old age, and short follow‐up times; implying that more attention should be paid to these populations. The potential for higher incidence of AD in patients with AF, although has been corrected for covariates not adequately balanced in Table 3, it could be the case that AF and AD are two potential manifestation of possible variables not considered in the present study, without any pathophysiological relation.

AD, which might involve coronary injury, pericardial involvement, and other direct cardiac effects, could increase the risk of AF.^3^, ^4^, ^5^, ^6^, ^7^, ^8^, ^9^ However, there is no argument made in support of an association between AD and subsequent AF although several molecular mechanisms involving the atrial remodeling and weakness of the aortic walls might be possible explanations.^15^, ^16^, ^17^, ^18^ Such findings based on this big dataset deserved further investigation.

## 5 LIMITATIONS

First, the Taiwanese NHIRD has the power of large numbers, but it does not provide additional physiological insight. Second, diagnoses were retrieved from only inpatient files. This might introduce a bias, as AF patients with concomitant disorders might more often be hospitalized. Third, information about treatment was not collected in this study and this might have influenced the occurrence of both diseases and represents a possible bias in the interpretation of the results. Fourth, although we have used propensity matching and then conducted a multi‐variable analysis, it should be mentioned that uncontrolled potential confounders could be an issue in this type of study. Finally, the diagnostic accuracy of the diseases using ICD codes might be potentially the major limitation of the present study. However, this nationwide database has been validated and high accuracy was guaranteed.^19^, ^20^, ^21^, ^22^

## 6 CONCLUSION

This is a study evaluating the relationship between the presence of AD and the incidence of AF and vice versa in a large numbers of patients from Taiwan. A positive association for both was found in this study.

## CONFLICT OF INTEREST

The authors declare no potential conflict of interests.
